# The common interests of health protection and the economy: evidence from scenario calculations of COVID-19 containment policies

**DOI:** 10.1007/s10198-022-01452-y

**Published:** 2022-03-19

**Authors:** Florian Dorn, Sahamoddin Khailaie, Marc Stoeckli, Sebastian C. Binder, Tanmay Mitra, Berit Lange, Stefan Lautenbacher, Andreas Peichl, Patrizio Vanella, Timo Wollmershäuser, Clemens Fuest, Michael Meyer-Hermann

**Affiliations:** 1 ifo Institute – Leibniz Institute for Economic Research at the University of Munich, Poschingerstr. 5, 81679 Munich, Germany; 2 Department of Systems Immunology and Braunschweig Integrated Centre of Systems Biology, Helmholtz Centre for Infection Research, Rebenring 56, 38106 Braunschweig, Germany; 3 Department of Epidemiology, Helmholtz Centre for Infection Research (HZI), Inhoffenstr. 7, 38124 Brunswick, Germany; 4 German Center for Infection Research (DZIF), Inhoffenstr. 7, 38124 Braunschweig, Germany; 5 Institute for Biochemistry, Biotechnology and Bioinformatics, Technische Universität Braunschweig, Braunschweig, Germany; 6 Economics Department, University of Munich, Ludwigstr. 28, 80539 Munich, Germany; 7 CESifo Munich, Poschingerstr. 5, 81679 Munich, Germany; 8 Chair of Empirical Methods in Social Science and Demography, University of Rostock, Ulmenstr. 69, 18057 Rostock, Germany

**Keywords:** COVID-19, Optimal strategy, Economy, Deaths, Integrated simulations, Real-time analysis

## Abstract

We develop a novel approach integrating epidemiological and economic models that allows data-based simulations during a pandemic. We examine the economically optimal opening strategy that can be reconciled with the containment of a pandemic. The empirical evidence is based on data from Germany during the SARS-CoV-2 pandemic. Our empirical findings reject the view that there is necessarily a conflict between health protection and economic interests and suggest a non-linear U-shape relationship: it is in the interest of public health and the economy to balance non-pharmaceutical interventions in a manner that further reduces the incidence of infections. Our simulations suggest that a prudent strategy that leads to a reproduction number of around 0.75 is economically optimal. Too restrictive policies cause massive economic costs. Conversely, policies that are too loose lead to higher death tolls and higher economic costs in the long run. We suggest this finding as a guide for policy-makers in balancing interests of public health and the economy during a pandemic.

**Supplementary Information:**

The online version contains supplementary material available at 10.1007/s10198-022-01452-y.

## Introduction

The SARS-CoV-2 pandemic confronts the world with a rapid spread of infections and deaths associated with COVID-19. Several governments have used or are still using non-pharmaceutical interventions (NPIs) such as social distancing regulation, prohibition of public events, school closures, or restrictions of business activity to slow down and contain the pandemic. Evidence suggests that these measures indeed reduce the number of infections [1–3]. At the same time, the pandemic and shutdown measures give rise to substantial economic costs [4, 5].

In the public debate, interests of public health and the economy are often presented as being in conflict [6, 7]. Although this trade-off view may seem intuitive, evidence on medium- and long-run economic consequences of past epidemics suggests that an unregulated spread of a virus with larger disease burden can also have adverse effects on the economy [8–10]. New infection waves, e.g., due to accelerated loosening of restrictions, could cause a large rise in absenteeism from work due to illness and could reduce trust of consumers and investors. As consequence, companies would have to shut down or to reduce their business activities again—regardless of government regulations—resulting in considerable further costs. Conversely, stricter regulations may also give rise to indirect disease burden in other areas [11]. The aim is to make the fight against the pandemic sustainable and to reconcile public health and economic objectives [12].

An increasing number of studies on NPI strategies concludes that immediate shutdowns and health policy interventions is the most favorable strategy [10, 13–16]. A separate question in the public debate, however, is about the optimal shutdown duration, and the timing and speed of the phasing-out of NPIs [17, 18]. A conflict between health protection and economic interests arises if a strategy with lower economic costs leads to significantly higher death numbers. Such a conflict would be particularly challenging if the reduction of economic costs requires a rapid opening process. Yet, previous studies using integrated macroeconomic and epidemiological models conclude that limiting the spread of the virus is the economically optimal reopening policy [19–22]. We add to this literature by examining economically optimal exit strategies that can be reconciled with public health.

We provide a novel simulation approach integrating epidemiological and economic models that allows data-driven real-time analysis during a pandemic. Using data on infection dynamics and industry-specific economic activity during the first shutdown in Germany in spring 2020, our simulation results suggest that it can be advantageous for both health and the economy to keep the effective reproduction number of infections well below one. We find that economic costs as a function of the reproduction number follow a U-shape: both an extensive opening strategy as well as a further tightening of the measures would have led to higher economic costs compared to a prudent opening strategy with a reproduction number of around 0.75.

Our results are based on a particular time and country—SARS-CoV-2 epidemic in Germany during spring 2020—with a given set of NPIs that have been implemented. Our quantitative results should be seen in this context, and the optimal strategy in other pandemics, time periods or countries may be different. But the qualitative conclusion remains: our study shows that public health and the economy are not necessarily in conflict, with a non-linear relationship between the reproduction number of the virus and economic output. It is in the interest of the economy to balance NPIs in a manner that keeps the epidemic under control and further reduces the incidence of infections. Conversely, policies that are too loose also lead to higher economic costs in the long run. We suggest this finding as an orientation for policy-makers in balancing interests of public health and the economy during a pandemic.

## Methods

### Using Germany as a case study

**Fig. 1:**
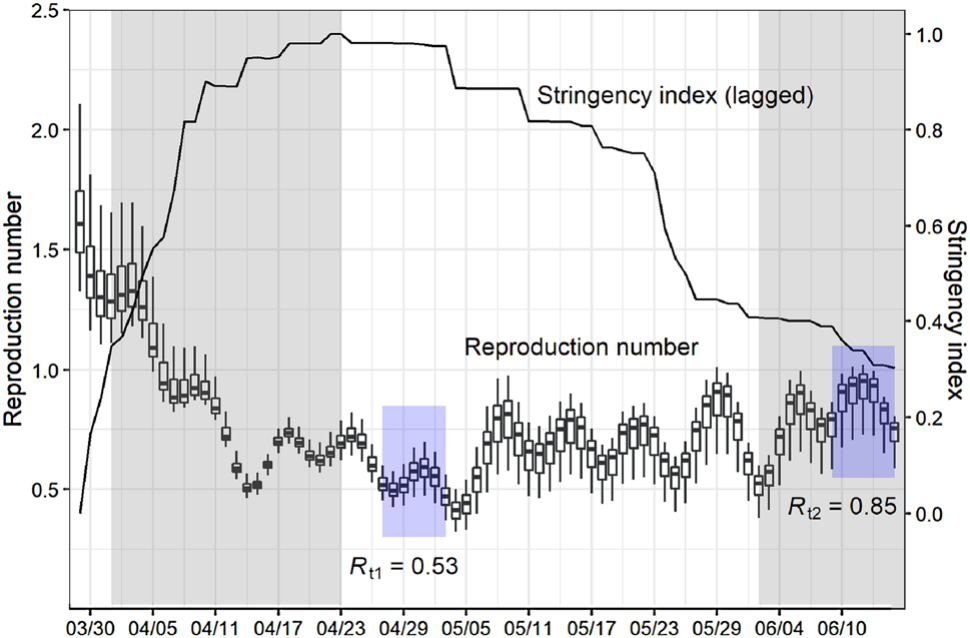
Reproduction number and NPI stringency in Germany. The boxplots illustrate the distribution of the $R_\mathrm{t}$ estimates for each date (median, 25 and 75 percentile) following the model described in *SI Appendix, pp. 4–10*. The error bars denote 1.5 times the interquartile range. The stringency index is given as the first principal component from a principal component analysis based on all NPIs on the federal state level in Germany and is plotted with a lag of 2 weeks. Information on the NPIs are taken from the Corona Data Platform (https://corona-datenplattform.de), released by the Federal Ministry for Economic Affairs in Germany. The shaded grey areas indicate the survey periods for the ifo Business Survey in April and June 2020. The shaded blue areas indicate the time window that was used to calculate the reference $R_\mathrm{t}$ values in the status quo before and after the impact of gradual lifting of NPIs

We use the first COVID-19 wave and shutdown in Germany to calibrate our models. Germany’s strategy during the first wave in spring 2020 with comparatively few deaths and low economic losses compared to other countries was discussed as a best practice example from an international perspective [23]. Germany introduced several restrictive measures in March 2020 to contain the spread of the virus: the NPIs included travel restrictions, restrictions on gatherings, the cancellation of events, the closure of schools and universities, hotels, bars and restaurants, the recommendation of home-office and hygiene rules, as well as the ordered shutdown of several social service providers and the stationary retail industry (excluding grocery stores). Some federal states introduced curfews. The sum of these measures—and of behavioral adjustments—reduced the spread of the virus. Figure 1 plots the time-dependent reproduction number, $R_\mathrm{t}$, and an NPI stringency index (the latter plotted with a lag of 14 days to account for the delayed impact of NPIs on the reproduction number). The stringency index is constructed from principal component analysis of all NPIs on the federal state level [24]. Figure 1 shows that, with a lag of 2 weeks, the stringency of Germany‘s first shutdown appears to inversely track $R_\mathrm{t}$. The reproduction number fell well below one in April, and the number of daily new reported cases decreased noticeably during the shutdown. On April 20${\text {th}}$, a gradual loosening of the restrictions was announced. With a lag of 2 weeks, $R_\mathrm{t}$ increased at the beginning of May.

### Combining methods from epidemiology and economics to estimate the impact of reopening

The status quo of our scenario calculations represents the situation of $R_\mathrm{t}$ and economic activity in the initial shutdown phase until the gradual opening process started on April 20${\text {th}}$. Starting from the status quo, we simulate various scenarios for a further loosening or tightening of the shutdown measures. We model the death toll and economic activity as a function of $R_\mathrm{t}$, using an empirical relationship between $R_\mathrm{t}$ and activity at the industry sector level as well as the time until the economy fully recovers. In the model, different shutdown policies are associated with different $R_\mathrm{t}$ values; more relaxed (restrictive) restrictions yield larger (smaller) $R_\mathrm{t}$ values, implying a longer (shorter) period until the containment of the epidemic is completed. A longer period due to more relaxed restrictions is associated with larger death tolls but also with higher economic activity in the short run. However, larger $R_\mathrm{t}$ values imply that daily new infections decrease slowly (or increase) and the time until full opening of the economy is extended.

Our scenario calculations are based on a novel and unique combination of epidemiological and economic simulation models (see *SI Appendix* for detailed description). The models are connected in two ways. First, we associate the $R_\mathrm{t}$ estimates from the epidemiological model with corresponding economic activity levels in different industries for two time periods. The first time period refers to the end of the initial shutdown, and the second to the period after the implementation of several step-by-step relaxations of NPIs. That way we estimate slopes for the (linear) relationship between the severity of shutdown restrictions ($R_\mathrm{t}$) and the corresponding activity levels for each industry. Second, the epidemiological model yields the estimated duration until the epidemic situation allows the full opening, which marks the beginning of a recovery phase in the economic model.

### The reproduction number depends on the severity of shutdown restrictions

In a first step, we employ a mathematical-epidemiological model with Susceptible-Exposed-Carrier-Infected-Recovered (SECIR) components to estimate the development of $R_\mathrm{t}$ in Germany (see Fig. 1, [3], and SI Appendix). The estimates are based on a dynamic adaptation of the model parameters to the incidence reporting database of the Robert Koch Institute (RKI), the German government’s central scientific institution for monitoring the situation on SARS-CoV-2. We specify $R_\mathrm{t1}=0.53$ as reference value that refers to the estimated reproduction number in Germany just before the partial lifting of the NPIs on April 20${\text {th}}$ (Fig. 1, left blue area). Similarly, we specify $R_\mathrm{t2}=0.85$ as reference value that refers to the reproduction number after the partial lifting, thus capturing the effect of lifting the NPIs on $R_\mathrm{t}$ (Fig. 1, right blue area).

### Severity of shutdown determines time until control of epidemic allows full reopening

A key assumption in our analysis is that reducing the number of new infections to 300 reported cases per day (corresponding to an incidence of 2.5 infections per 100,000 inhabitants in 7 days), would allow to fully control the epidemic through contact tracing and isolation by the approximately 400 public health offices in Germany. The remaining restrictions limiting economic activities could be completely lifted thereafter.

**Fig. 2:**
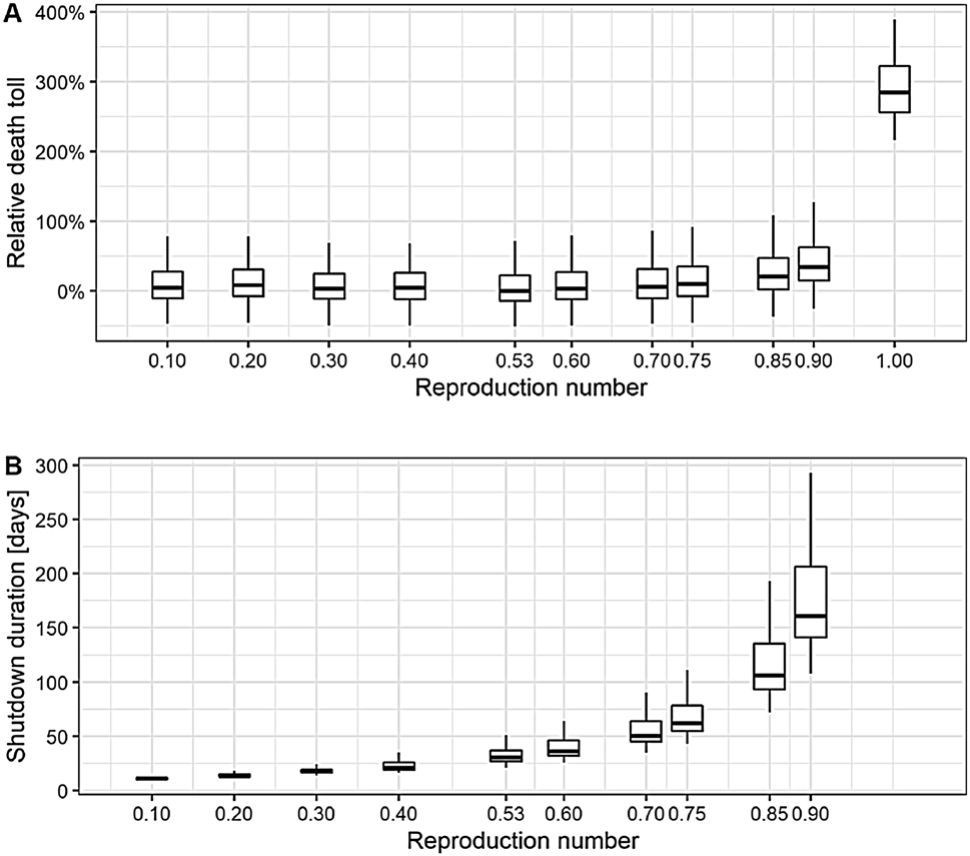
**A** Estimation of the relative death toll accumulated between April 20${\text {th}}$, 2020 and July 31${\text {st}}$, 2021 with the epidemic model, in percentage difference to the median value in the reference scenario ($R_\mathrm{t} = 0.53$). The reproduction number on the abscissa was fixed in the simulation from April 20${\text {th}}$, 2020 until reaching 300 daily new cases per day and then set to one. **B** Estimation of the shutdown duration needed to reach 300 new reported cases per day for each fixed reproduction number, starting on April 20${\text {th}}$. The boxplots illustrate the distribution of the estimates (median, 25 and 75 percentile). The error bars denote 1.5 times the interquartile range

In a prospective study, we assume different values of $R_\mathrm{t}$ to reflect the severity of the shutdown restrictions and keep these values constant in the respective scenarios until the threshold of 300 daily new infections per day is reached, determining the duration of the shutdown. The fewer restrictions are imposed, the longer the restrictions need to be kept in place to reach the threshold (Fig. 2B). Thus, a larger reproduction number delays a control of the epidemic. Importantly, the reproduction number impacts the period required to reach the threshold non-linearly. In addition, we consider a scenario where $R_\mathrm{t}$ is kept at one until a vaccine is available at large scale to vaccinate all relevant groups. In the baseline scenario, we assume that the vaccine becomes available at a large-scale on July 31${\text {st}}$, 2021.

### Economic activity depends on shutdown restrictions and speed of full reopening

In a second step, we integrate the results from the SECIR model into the economic model and simulate the economic costs at the industry level for different policy scenarios. The costs of a scenario are given as the aggregated loss of economic activity during the shutdown and economic recovery period after full reopening.

**Fig. 3:**
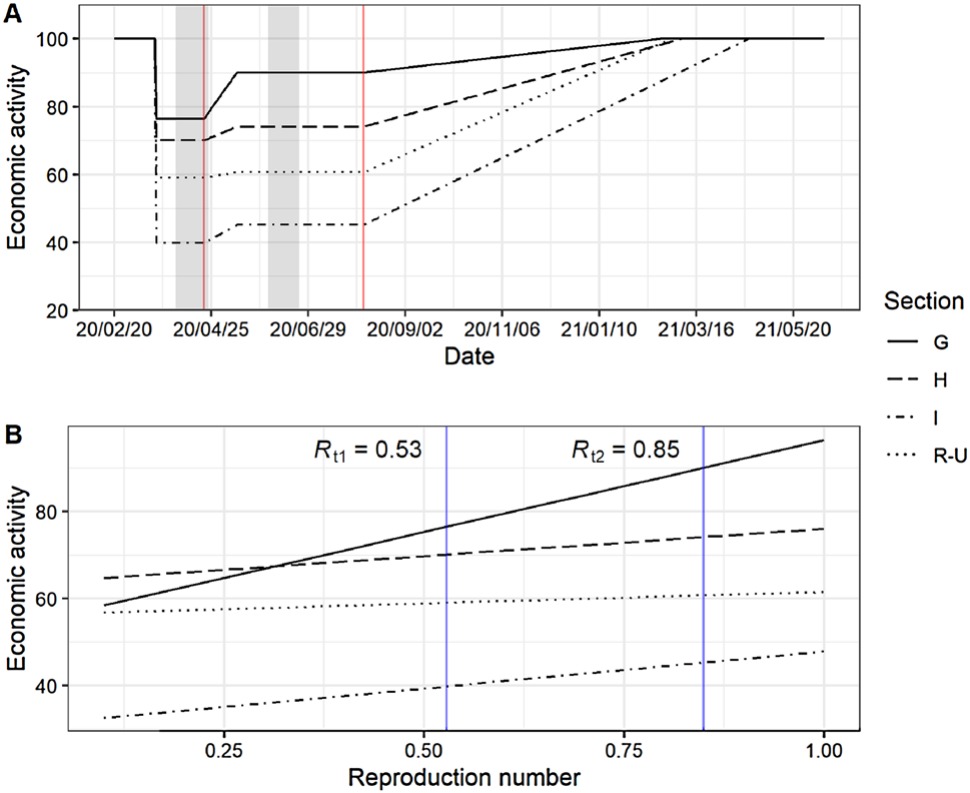
**A** The process of economic activity for the scenario where the policy-makers increase $R_\mathrm{t}$ from 0.53 to 0.85. Starting from the pre-shutdown level (normalized to 100), the economy experiences a decline in activity during the shutdown. On April 20${\text {th}}$, the policy-makers initiated a gradual lifting of NPIs (indicated with the first vertical red line). After the 300 daily new cases have been reached, the measures are lifted and the economy enters the recovery phase (indicated with the second vertical red line). The beginning of the recovery phase depends on the $R_\mathrm{t}$ value and the associated time period in Fig. 2B). Depicted are the activity levels for the economic sections *G* (wholesale and retail trade), *H* (transportation and storage), *I* (accommodation and food service activities), and *R* to *U* (entertainment and other service activities). The shaded grey areas indicate the survey periods for the ifo Business Survey in April and June. A more in-depth description of the model can be found in the supplement (see Fig. S4). **B** The linear relationships between changes in industry-specific economic activity and changes in the reproduction number. The vertical blue lines indicate the $R_\mathrm{t}$ values 0.53 and 0.85 that are used to estimate the slope

Figure 3A illustrates the process in the economic model for the scenario where the policy-makers tolerate an increase of $R_\mathrm{t}$ from 0.53 to 0.85 on April 20${\text {th}}$. This refers to the change in the reproduction number and economic activity that was empirically observed over that period of time in Germany. The model assumes that the economy starts from a pre-shutdown activity level and experiences a decline due to the shutdown imposed in March 2020. Activity levels of different industries in response to the shutdown are determined by the status quo before the exit process started on April 20${\text {th}}$. Prior to reaching the required 300 new cases per day allowing full reopening, our model assumes that policy-makers can decide on the further course of severity of restrictions in a period of partial opening. Loosening restrictions would ceteris paribus increase economic activity in the partial opening phase (see Fig. 3A). However, it would also give rise to higher $R_\mathrm{t}$ values, thus increasing the duration of this phase (see Fig. 2B). Conversely, tightening restrictions would lead to a reduction in economic activity, but reduce $R_\mathrm{t}$ and the time needed until the number of new infections would allow a full opening. The economy slowly recovers once all shutdown measures can be fully lifted without jeopardizing the containment of the epidemic because either a sufficiently low number of new infections is reached or a vaccine is available. At the end of the recovery phase, the economy returns to its pre-crisis activity level.

### Estimates of economic activity during shutdown

The estimates of economic activity are based on the ifo Business Survey, a monthly survey that includes roughly 9000 responses from German firm managers in manufacturing, the service sector, trade, and construction [25, 26]. Managers base their responses predominantly on information derived from their own business activities [26]. During the pandemic, this information also includes potential health-related issues, e.g., if there was a negative effect of infections on the workforce. We use the firms’ assessment of their current business situation as it is highly correlated with the gross value added and several official economic activity measures (see *SI Appendix, Tab. S3 and Fig. S5*) [27].

We relate firm responses during the survey periods to different shutdown and partial opening periods as well as the corresponding $R_\mathrm{t}$ values from the SECIR model (see Fig. 1). Specifically, we assume a linear relationship between $R_\mathrm{t}$ and changes in economic activity for each industry using the April and June surveys and the corresponding reference reproduction number values, $R_\mathrm{t1}=0.53$ and $R_\mathrm{t2}=0.85$. The April survey captures the activity levels in each industry during the shutdown before partially lifting restrictions, whereas the June survey captures the activity levels after the lifting process (see *SI Appendix, Fig. S2*).

### Heterogeneity and industry-specific impact of shutdown strategies

Figure 3B shows the industry-specific linear relationship between the reproduction number and economic activity, and illustrates that elasticities in economic activity are heterogeneous across industrial sections. For example, lifting shutdown restrictions gives rise to a larger increase of economic activity in retail (*G*) compared to transportation industries (*H*). Moreover, gradual lifting of measures was more restrictive for the entertainment industries (e.g., events) and other social service activities (*R*–*U*).

Not all industries in Germany were directly affected by legal shutdown measures. The manufacturing industry or electricity firms, for example, were not included in any state order to shut down their business activities in Germany. However, these industries also experience large slumps in activity because of declined domestic and foreign demand, disrupted supply chains, or absences from work due to illness and/or quarantine. In our simulation model, we thus distinguish between exogenous and endogenous industries (see *SI Appendix, Tab. S2*). The former refer to the industries that are exogenously shut down by the government and are treated as illustrated in Fig. 3B. These include, among others, firms in retail trade, accommodation and food services, transportation, entertainment and recreation, and several other social service activities. Changes in activity levels in endogenous industries such as the manufacturing sector, however, are not closed by shutdown measures but affected by changes in the economic output of the treated exogenous industries. We exploit inter-industry linkages and use input–output tables for the German economy to specify to what extent the activity level in one industry is affected by changes in other industries [28]. That way our approach only considers changes in economic activity of the endogenous industries that are driven by changes in treated (exogenous) industries.

We introduced a special question in the ifo Business Survey where respondents were asked about the expected duration until their business situation would return to normal once all shutdown measures are lifted. For the reference scenario ($R_\mathrm{t} = 0.53$), we take the mean of these expectations for each industry to calibrate our model (see *SI Appendix, Tab. S4*). We assume that it takes the firms two months less to fully recover in the scenario with $R_\mathrm{t} = 0.85$ compared to the reference scenario. That way we construct a data-based linear relationship between $R_\mathrm{t}$ and the time to recover to the pre-crisis level for different industries (see recovery phase in Fig. 3A).

In a globalized world, changes in NPIs and economic developments in other countries may affect domestic export-oriented industries. The manufacturing industry in Germany, which contributes about a quarter to the economic output, is particularly affected in that respect [29]. We implicitly control for the effect of the developments abroad by holding the shutdown and recovery duration for endogenous sectors constant across our domestic policy scenarios. By doing so, any effects from other countries on export-oriented industries are the same in each policy scenario. Industries treated by our domestic policy scenarios, by contrast, only have a minor share in German exports (see *SI Appendix, Fig. S7*). As such, the *relative* comparison of scenarios’ costs are robust to other countries’ developments.

## Results

### Estimated COVID-19-associated death toll

Starting from the partial easing of NPIs after the first shutdown in Germany on April 20${\text {th}}$, the calibrated simulation model projects the number of expected additional COVID-19 deaths until July 31$\mathrm{st}$, 2021. The death toll rises with increasing reproduction numbers, although the differences are relatively small up to $R_\mathrm{t}=0.75$ and stay within a range of 10% additional deaths relative to the reference scenario with $R_\mathrm{t}=0.53$ (Fig. 2A). In contrast, the death toll rises sharply from $R_\mathrm{t}=0.9$ onward due to non-linearities. Expected additional deaths increase to around 300% compared to the reference scenario when following a policy at $R_\mathrm{t}=1.0$.

### The long-term economic costs are minimal at intermediate reproduction numbers

**Fig. 4:**
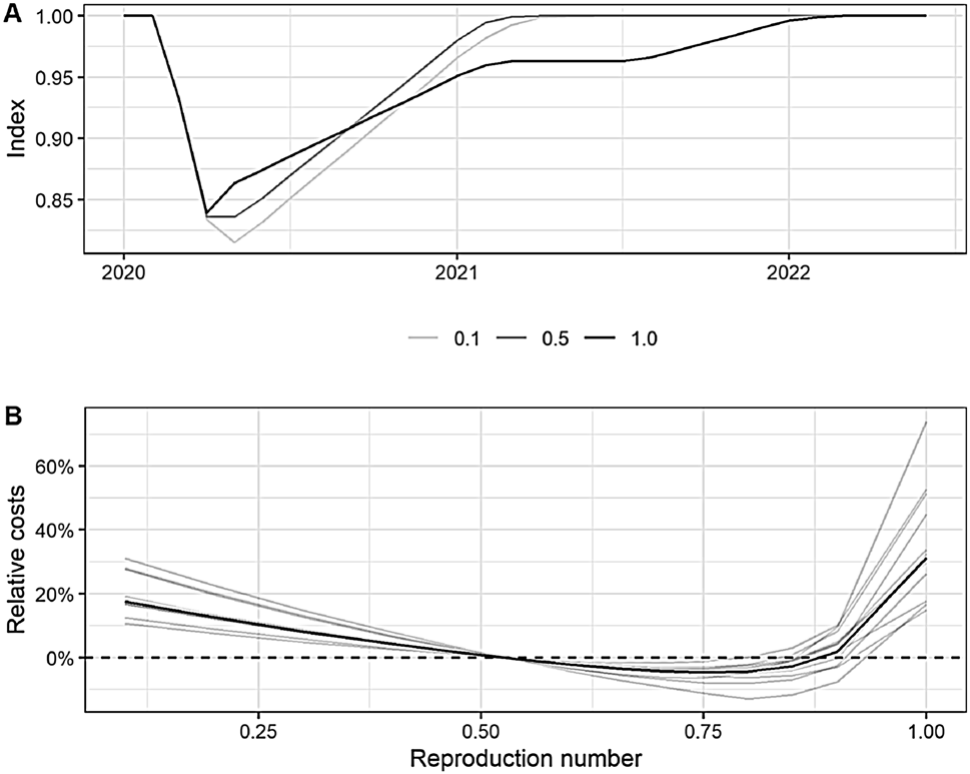
**A** Overall economic activity over time for three baseline policy scenarios (denoted by their respective reproduction numbers, 0.1, 0.5, and 1.0). Pre-crisis economic activity is normalized to 100. **B** Relative costs for each policy scenario, in percentage difference to the reference scenario ($R_\mathrm{t} = 0.53$). Economic costs are given as the aggregated loss of activity occurring as a result of the shutdown and recovery phase. The bold line indicates the baseline scenarios; the shaded grey lines indicate the results of the robustness tests. The numeric values can be found in the *SI Appendix, Tab. S3*

The total economic costs of the scenarios result from the aggregated loss of economic activity among all industries over the years 2020–2022. Figure 4A shows the development of aggregated activity in our policy scenarios as deviations from the pre-crisis activity level (normalized to 100). For instance, the scenario with $R_\mathrm{t} = 0.1$, i.e., where the policy-makers further intensify the shutdown, would further reduce economic activity. On the other hand, lifting the restrictions such that the reproduction number increases to one gives rise to larger economic activity thereafter. However, some restrictions have to be kept in place for such a long time that the pre-crisis economic activity level is not reached before 2022. Figure 4B shows the relative costs for all scenarios compared to the reference scenario ($R_\mathrm{t} = 0.53$). The relative costs are given as the percentage differences in total loss of economic activity. The results show that both a strategy with extending high levels of restrictions ($R_\mathrm{t} < 0.53$) and a strategy with too aggressive loosening of measures ($R_\mathrm{t} > 0.9$) would lead to higher relative economic costs. Compared to the strategy of keeping restrictions of the initial shutdown period ($R_\mathrm{t}=0.53$), costs decrease in a strategy of a slight loosening of restrictions. The long-term economic costs are minimal if the reproduction number is around 0.75.

### Common interest of economy and health

We cannot identify a conflict between the economy and health protection in relation to a strong relaxation—the costs would be higher in both dimensions. Accelerated opening leads to substantially more COVID-19 deaths and increased economic costs. Our findings clearly challenge statements which suggest exit strategies with $R_\mathrm{t}$ values close to one to be economically preferable [21]. While strong opening policies would allow for more economic activity in the short term, our simulations suggest that the long duration of remaining restrictions would increase relative economic costs compared to alternative gradual opening strategies.

Our results suggest that a balanced strategy is in the common interest of health protection and the economy. The scenario calculations show that a slight, gradual lifting of shutdown restrictions which keeps reproduction numbers at an intermediate level and which allows to further reduce infection numbers in a significant manner is suitable to reduce the economic losses without jeopardizing medical objectives.

### Robustness of results suggests general applicability

Clearly, generalization of our results beyond Germany and across time is limited to comparable regions, situations and given NPIs. The relationship between economic activity and the reproduction number might not be the same across world regions. Moreover, the shutdown duration and final death toll are influenced by the number of new infections at the point of entering or changing shutdown measures. However, our results are robust to several sensitivity tests in assumptions regarding the relationship between the shutdown severity and economic activity, affected industries of exogenous shutdown restrictions, the duration of economic recovery, and the number of daily new cases that needs to be reached to control the epidemic. We also tested the sensitivity of our assumption on the time of large-scale availability of a vaccine (see *SI Appendix, Tab. S5*).

The assumption of a linear relationship between shutdown levels and economic activities is clearly a simplification in our simulation model, although the slope of our linear relationship is based on observed data. Our robustness tests include simulations with (non-linear) isocost-curves that indicate how severely the linear assumption needs to be violated for our results to no longer be valid. The results show that it would require implausible assumptions of extreme non-linearities to invalidate our findings (see *SI Appendix, Fig. S6*). All robustness tests can be found in the supplement (*SI Appendix, Tab. S5*). Our inferences do not change. Minima of relative economic costs are between $R_\mathrm{t}$ values of 0.7 and 0.8 in all sensitivity tests (see light-grey lines in Fig. 4B).

## Discussion

We consider the qualitative statement of our results to be robust and of general nature. It is in common interest of health and the economy to implement opening policies with prudent steps and to closely monitor the respective reaction of the infection figures. Our conclusion is in line with retrospective studies of the influenza epidemic in 1918 in the USA [13, 30], and current economic studies supporting a strategy to manage the COVID-19 pandemic [19, 20]. We show that it is also in the interest of the economy to balance non-pharmaceutical interventions in a manner that further reduces the incidence of infections. By contrast, NPI policies that are too loose could cause higher economic costs in the long term. We provide an additional guideline for policy-makers whether extending or easing restrictions minimizes long-term economic costs once the effective reproduction number is already below one. Using counteracting measures—such as face masks, behavioral rules, improved trace and isolation techniques, new technologies and increased testing—may limit the spread of the virus or even may help to contain the pandemic [16, 31–36] and thus creates leeway for larger opening and economic recovery. The level of economic restrictions thus depends to a large extent on technical improvements and behavioral adjustments of the population until a vaccine or effective medical treatment is available at large scale for all in need.

## Supplementary Information

Below is the link to the electronic supplementary material.

Supplementary file 1 (pdf 2206 KB)
