# Supplementary material for: The common interests of health protection and the economy: evidence from scenario calculations of COVID-19 containment policies
Source: Eur J Health Econ. 2022 Mar 19;24(1):67–74. doi: 10.1007/s10198-022-01452-y (PMC8934060; doi:10.1007/s10198-022-01452-y)
Supplement: Supplementary file 1 — (pdf 2206 KB) [file 10198_2022_1452_MOESM1_ESM.pdf]

Supplementary Information (SI) for

The common interests of health protection and the economy: Evidence from scenario calculations of COVID-19 containment policies

This PDF file includes:

Supplementary text

Figs. S1 to S7 and Tables S1 to S5

SI References

Supporting Information Text

Background: Containment policy in Germany during the first shutdown

In order to contain the spread of the COVID-19 epidemic in Germany, the national and federal state governments introduced restrictive measures in several stages since March 2020. These included the banning of major events, the closure of schools and day-care centres, the forced shutdown of numerous companies in the retail, catering and many (social) services sectors, and the introduction of mobility and contact restrictions (with more or less strict curfews). In addition, many companies reduced their business activities in order to protect their employees or due to reduced demand. Citizens also adapted their behavior due to the new risk situation and information policies. The sum of these measures and behavioral changes appears to have influenced the reproduction rate in Germany: R_t fell well below one and the number of registered new infections per day decreased during the shutdown (see Fig. S2). At the same time, however, the consequences of the COVID-19 pandemic and the shutdown measures have plunged the German economy in Q2/2020 into what is by far the deepest recession in its post-war history (1).[*] Since the German containment strategy in the spring SARS-CoV-2 wave seems to have been successful and the feared scenarios of casualties in Germany have so far failed to materialise, a growing public community called for faster loosening of restrictions in all areas. On April 20th 2020, a conference of the national and state governments agreed on a gradual, step-by-step loosening of the shutdown measures.[†]

Materials and Methods

Mathematical-epidemiological model. The development of infection dynamics has been addressed by a number of mathematical approaches based on differential equation models (7) and agent-based models (8). Our SECIR model is a deterministic ordinary differential equations (ODEs)-based model in which different stages of the infection and associated viral spreading are considered. The model structure and parameter ranges were chosen according to the specific properties of SARS-CoV-2 viral infections. The model comprises of compartments representing the individuals susceptible (S) or exposed (E) to the virus, asymptomatic carriers ($C_{\mathrm{I,R}}$) which may become symptomatic ($I_{\mathrm{H,R,X}}$), hospitalized ($H_{\mathrm{U,R}}$) or in the need of intensive care $U_{R,D}$. Infected individuals have terminal fate of recovery ($R_{\mathrm{Z,X}}$) or death (D). The carrier compartments refer to an infection state of an individual without symptoms who is able to transmit the disease to susceptible individuals, and later may or may not develop any symptoms. In this state, the transmissibility of the virus can be significant as the individual is not aware of the disease and could actively make contact with susceptible individuals. Therefore, the presence of such a compartment is crucial in the models for SARS-CoV-2 (9). A schematic representation of the model is shown in Fig. S1A. The model equations read

[*] It can be assumed that an unhindered spread of the virus would also have been associated with very high economic and health costs.

[†] The actual death toll for Germany counts around 4,500 registered COVID-19 deaths on April 20th, around 6,500 two weeks later at the beginning of May, around 8,500 at the beginning of June, and around 9,000 at the beginning of July 2020. Germany has a population of around 83 million (2). The death toll corresponds to relative numbers of around 5.3 deaths per 100k inhabitants (April 20th), 10.2 (June 1st), and 10.8 (July 1st). In comparison, two months after the start of relaxations, relative death rates have been around seven times larger in the UK, around five times larger in Sweden, and around four times larger in the USA (3–6).

$$\frac{dS}{dt} = -R_1(t)\frac{(C_I + C_R + I_X + \beta(I_H + I_R))}{N}S, \tag{1}$$

$$\frac{dE}{dt} = R_1(t)\frac{(C_I + C_R + I_X + \beta(I_H + I_R))}{N}S - R_2E, \tag{2}$$

$$\frac{dC_I}{dt} = (1-\alpha)\, R_2E - R_3C_I, \tag{3}$$

$$\frac{dC_R}{dt} = \alpha R_2E \; - R_9C_R, \tag{4}$$

$$\frac{dI_H}{dt} = \mu\rho(t)R_3C_I - R_6I_H, \tag{5}$$

$$\frac{dI_R}{dt} = \mu\,(1-\rho(t))\, R_3C_I - R_4I_R, \tag{6}$$

$$\frac{dI_X}{dt} = (1-\mu)\, R_3C_I - R_4I_X, \tag{7}$$

$$\frac{dH_U}{dt} = \vartheta(t)R_6I_H - R_7H_U, \tag{8}$$

$$\frac{dH_R}{dt} = (1-\vartheta(t))\, R_6I_H - R_5H_R, \tag{9}$$

$$\frac{dU_D}{dt} = \delta(t)R_7H_U - R_{10}U_D, \tag{10}$$

$$\frac{dU_R}{dt} = (1-\delta(t))\, R_7H_U - R_8U_R, \tag{11}$$

$$\frac{dR_Z}{dt} = R_4I_R + \; R_5H_R + \; R_8U_R, \tag{12}$$

$$\frac{dR_X}{dt} = R_9C_R + \; R_4I_X, \tag{13}$$

$$\frac{dD}{dt} = R_{10}U_D. \tag{14}$$

The rates $R_{2,\ldots,10}$ denote the inverse time of transition between the respective states and were inferred from the literature. Parameter R_1 is fitted to the course of reported case numbers in a sliding time window and, therefore, is a time-varying parameter. Parameter β determines the interaction intensity of infectious symptomatic individuals (I_H and I_R) with the susceptible population. Parameters α, μ, ρ, ϑ and δ denote fractions of individuals toward a particular fate. A complete description of the parameters' definition and value sets is given in (10). Parameters ρ, ϑ and δ have a time-varying component modelled with a logistic function

$$\rho = \rho_0\sqrt[3]{k(t)/\bar{k}}, \quad \vartheta = \vartheta_0\sqrt[3]{k(t)/\bar{k}}, \quad \delta = \delta_0\sqrt[3]{k(t)/\bar{k}}, \tag{15}$$

$$k(t) = H - (H-L)\left(\frac{1}{1+e^{-k_0(t-t_0)}}\right), \tag{16}$$

where t corresponds to the time of the year starting from January 1st 2020, $\bar{k} = \rho_0\vartheta_0\delta_0$, $H = 0.156$, $L = 0.011$, $k_0 = 0.25$, and $t_0 = 89$ are obtained from fitting the case fatality rate (CFR) ($k(t) = \rho\vartheta\delta$) which changed over the course of the epidemic in Germany. This is due to changing testing frequencies and the shifting age structure of the infected over time (11); therefore, the CFR is not completely reflecting a change in the fatality rate of the virus. The uncertainty in the parameter values was incorporated into the analysis by repeated (100 times) random sampling within the plausible ranges and obtaining the distributions of model variables.

The basic reproduction number (R_0) is defined as the expected number of secondary cases produced by a single infection in a susceptible population and is a constant characteristic of viral dissemination dynamics in a passive community. However, the dynamics of the epidemic may be under the influence of multiple time-dependent factors such as change in the individual behavior in response to policies or public awareness. Therefore, a time-dependent reproduction number (R_t) describing the expected number of secondary cases per index case at a given time of the epidemic is a more relevant quantity for an active community and reflects the multi-factorial impact of non-pharmaceutical interventions (NPIs), behavioral changes, seasonal effects, etc. on the dynamics of viral spread.

Herein, the temporal evolution of R_t was obtained by re-fitting the model in a sliding time-window of the data which was shifted throughout the duration of the epidemic (see Fig. S1B). This method allows to adapt the model parameter R_1 that is associated with NPIs in order to fit the data, which cannot be achieved by a fixed parameter value. The value of R_t corresponding to the k-th time-window is calculated based on the fitted value of R_1 by

$$R_k = R_{1,k}\frac{S_k}{N_k}\left[\frac{1-\alpha}{R_3} + \frac{(1-\alpha)\left[\beta\mu\,(1-\rho_k) + (1-\mu)\right]}{R_4} + \frac{\beta\mu\,(1-\alpha)\,\rho_k}{R_6} + \frac{\alpha}{R_9}\right], \tag{17}$$

where $\rho_k := \rho(t_k)$ denotes the average value of the time-varying parameter and $R_{1,k}$ is the fitted value of R_1 in the k-th time-window, $S_k := S(t_k)$ and $N_k := N_0 - D(t_k)$ are related to the first time point in the k-th time-window, and N_0 is the total population at the beginning of the epidemics. The reproduction number in (17) is derived from the SECIR model (14) using the next generation matrix method (12, 13). The cumulative reported case number is compared to the sum of infected and detected individuals, i.e. with $I_H + I_R + H_U + H_R + U_R + U_D + D + R_Z$. The parameter R_1 is estimated for each time window of 7 days using Nelder-Mead simplex algorithm (14) (see Fig. S1B).

Plausible ranges of the parameters were estimated from the literature to account for their uncertainty (see Table S1). The parameter values were then randomly sampled within these ranges 100 times to derive a quasi-empirical distribution of the R_t value.

For simulating prospective dynamics of the epidemic from a defined starting date, the state condition of the model based on available retrospective data was calculated. Then, the development of model variables was obtained by imposing fixed R_1 values corresponding to different R_ts of interest (see Fig. S1C). The assumed prospective R_ts values can be linked to different degrees of strictness of the imposed measures.

Time until new infections are under control. Following Khailaie et al. (10), we assume that the approximately 400 health authorities in Germany have sufficient capacities to control 300 new cases per day through contact tracing and isolation. For the bundles of measures with varying severity, we calculated the time it takes to reach a maximum of 300 new reported cases per day (Fig. 2B) and the number of projected COVID-19 deaths (Fig. 2A). This was calculated by obtaining the daily influx of cases to the infected and recovering compartments I_H and I_R. The analysis was also repeated for capacities of 200 and 400 new cases per day in our robustness tests to reflect the sensitivity of the impact of different assumptions in the capacity of health authorities. Once the respective assumed threshold is reached, an R_t value of 1 and a targeted isolation of identified newly infected and their contacts ($\beta = 0$) were assumed. These assumptions keep the number of new infections around the target value. Alternatively, we have considered a scenario in which the current daily infection rates are kept constant until the earliest realistic availability date of a vaccine for the entire population, i.e. R_t is assumed to be at value of 1.

The calculated duration shall be interpreted as the further time necessary to retain the shutdown or restrictive measures. Based on the reduction in economic output in the various economic sectors on April 20th, 2020, and the R_t value corresponding to the shutdown period (i.e. in the status quo before the first relaxation of measures on April 20th, 2020), the costs of maintaining the shutdown or restrictive measures until reaching 300 (or 200 or 400) new reported cases per day were estimated for each assumed scenario with $R_\mathrm{t} < 1$. For the scenario of $R_\mathrm{t} = 1$, we assume that there will be restrictive measures until a vaccine becomes generally available. The Paul Ehrlich Institute[‡] estimated in spring 2020 that combined Phase II/III trials of a vaccine could start in autumn/winter 2020. First emergency approvals were indeed licensed at the turn of the year. However, it will take several more months until the vaccine will be available at a sufficient number. For our scenario, we therefore assumed this date to be July 31st, 2021. In our sensitivity tests, we assume the vaccine to be available at a large scale 120 days earlier or later compared to the baseline assumption.

NPIs and their impact on R_t. In order to quantify the impact of lifting measures, we assumed a 2-weeks time delay from a person being exposed to the virus to becoming symptomatic and reported in the database. For the first openings in Germany on April 20th, 2020, the reporting delay assumption implies that a person exposed on the first day of lifting measures will be reported on May 4th. However, as the R_t value reported on each date includes the impact of 6 other days in the data retrospectively (see Fig. S1B), the R_t value calculated on May 4th is biased by cases exposed before April 20th (6 out of 7 data-points). With a similar reasoning, the R_t values calculated in the period of May 4th-May 9th are contaminated with infected cases before April 20th, 2020. Therefore, the impact of the openings on April 20th shall be inferred from May 10th at the earliest. Since the obligation of wearing masks was imposed very shortly after the first openings, we considered them as a bundle of NPIs. Following the reporting delay assumption, the impact of the NPIs bundle on the R_t value is expected from May 19th at the earliest. In order to take into account the seasonality observed in the data and the R_t values in the calculation of NPI impacts, we considered a pooled set of R_t values in the 1-week period of May 19th-May 25th (see Fig. S2 and S3).

The R_t value corresponding to the complete shutdown (before April 20th) was calculated by excluding the contaminated period (May 4th-May 9th), following a similar reasoning. Therefore, we considered the R_t values at the latest possible week, the period of April 27th-May 3rd. The impact of the second nationwide lifting of measures was calculated by pooling the latest available week at the time of this analysis (see Fig. S2 and S3).

Economic model and empirical implementation.

Modelling economic costs. The economic costs of scenario s are given as the aggregated loss of activity occurring as a result of the shutdown. Denote y_m^s as the economic activity compared to the pre-shutdown level in scenario s and month m, with $0 \leq y_m^s \leq 100$. $y_m^s = 100$ refers to the pre-shutdown activity level, and $y_m^s = 0$ to an economy with zero production. Total costs of scenario s can be written as:

$$C^s = \sum_{m=1}^{M} 100 - y_m^s,$$

[‡] The Paul Ehrlich Institute is a German research institution and medical regulatory body, and is the German federal institute for vaccines and biomedicines.

where M is the time horizon under consideration, i.e. the total number of months that are taken into account in the analysis. Denote C^{ref} as the cost of a reference scenario. The relative costs of s are then given as $\Delta C^s = (C^s/C^{ref}) - 1$, such that $\Delta C^s > 0$, indicates scenarios with higher costs and lower aggregate economic activity compared to the reference.

The key challenge is to model y_m^s. We assume that $y_m^s = 100$ prior to the implementation of the measures, $0 \leq y_m^s \leq 100$ during the shutdown and the recovery phase, and $y_m^s = 100$ after the recovery phase. In other words, starting from the pre-shutdown activity level, activity drops during the shutdown, and recovers once the measures are lifted until the economy has returned to its pre-epidemic activity level.

Taking into account that the impact of the shutdown varies across industries, we explicitly model activity at the industry level. Denote $y_m^{s,j}$ as the activity for scenario s, month m, and industry j. y_m^s is then given as the average of each industry-specific activity, weighted by the share of the industry in total output, denoted as α_j:

$$y_m^s = \sum_{j=1}^{J} \alpha_j y_m^{s,j},$$

where J is the total number of industries in the economy.

For each s and j, the process of $y_m^{s,j}$ over m is modelled as follows. Denote $y_d^{s,j}$ as the activity on day d, with $0 \leq y_d^{s,j} \leq 100$ and $d \in \{1, \ldots, D\}$, where D is the last calendar day in the last year of observation. $y_m^{s,j}$ is given as

$$y_m^{s,j} = \frac{\sum_{d=1}^{D} y_m^{s,j} I_d^m I_d^w}{\sum_{d=1}^{D} I_d^m I_d^w} \times 100,$$

where I_d^m is an indicator variable equal to one if calendar day d belongs to calendar month m, and zero otherwise. Similarly, I_d^w indicates whether the calendar day is a working day or not (i.e. whether it falls on a weekend or a public holiday). This notation implies that the distribution of holidays across the calendar year is relevant for the cost of a scenario. That is, the shutdown is less costly when it is in place during months with few working days.

Furthermore, denote B as the calendar day when shutdown measures were implemented first, S as the day when a new policy is introduced (changing the severity of the measures), and $R^{s,j}$ as the day when the measures are lifted. $R^{s,j}$ is determined by the epidemiological model and describes the calendar day during which a certain daily case number has been reached. The superscripts indicate that there is heterogeneity across scenarios and industries. After the introduction of the new policy at S, the economy adjusts over the period $s^{s,j}$, after which the new activity level is reached. After the prescribed number of new infections is reached and the shutdown is fully lifted, i.e. for $d > R$, the economy slowly recovers and returns to its pre-shutdown activity. We assume that economic activity increases linearly from S to $S + s^{s,j}$ and from $R^{s,j}$ to $R^{s,j} + r^{s,j}$. $r^{s,j}$ denotes the industry-specific duration of the recovery period (in calendar days). For each day d, the activity in each scenario s and sector j is then given as follows:

$$y_m^{s,j} = \begin{cases} 1 & \text{for } d < B \\ y^{b,j} & \text{for } B \leq d < S \\ y^{b,j} + (d - S + 1)\frac{y^{s,j} - y^{b,j}}{s^{s,j}+1} & \text{for } S \leq d < S + s^{s,j} \\ y^{s,j} & \text{for } S + s^{s,j} \leq d < R^{s,j} \\ y^{s,j} + (d - R^{s,j} + 1)\frac{1 - y^{s,j}}{r^{s,j}+1} & \text{for } R^{s,j} \leq d < R^{s,j} + r^{s,j} \\ 1 & \text{for } d \geq R^{s,j} + r^{s,j} \end{cases}$$

$y^{b,j}$ and $y^{s,j}$ refer to the activity after the first introduction of shutdown measures and after the policy change, respectively. Figure S4 illustrates the process of $y_d^{s,j}$ over d.

Implementing the economic model. Implementing the economic model requires estimates for $y^{b,j}$, $y^{s,j}$, $R^{s,j}$, and $r^{s,j}$. B, S, and $s^{s,j}$ are determined exogenously. For our application, we specify March 19th, 2020 as the introduction date of the shutdown measures (i.e. $B = 79$). S refers to April 20th, when the national and state governments agreed on a gradual, step-by-step loosening of the shutdown measures (i.e. $S = 111$). $s^{s,j}$ is set to 21. That is, we assume that it takes an industry three weeks to adjust and reach the new activity level. The specification of $s^{s,j}$ is arguably ad-hoc, but the results are robust to different specifications. $R^{s,j}$ is estimated with the SECIR model and refers to the number of days until the infection numbers allow full opening. The recovery speed depends on the reproduction number R_{t}. In general, a higher (smaller) R_{t} value is associated with a higher (smaller) $R^{s,j}$,

Estimates for $y^{b,j}$, $y^{s,j}$, and $r^{s,j}$ are obtained from the ifo Business Survey, a long-running monthly panel survey of roughly 9,000 German firms (15) which covers the most important industries of the German economy as defined by the NACE Rev. 2 classification.[§] Economic activity, $y^{b,j}$ and $y^{s,j}$, is approximated by the companies' assessment of their own current business situation, which they can describe as "good", "satisfactory", or "poor". According to a meta survey, the information used by managers to assess their business situation is mainly firm-specific (16). Respondents of the ifo Business Survey view their profit

[§]The survey excludes public services (public administration, defence, compulsory social security, education, human health and social work activities) summarized by sections O, P and Q, as well some small industries which play virtually no role for economic fluctuations (A: agriculture, forestry and fishing; B: mining and quarrying; D: electricity, gas, steam and air conditioning supply; E: water supply, sewerage, waste management and remediation activities; K: financial and insurance activities; U: activities of extraterritorial organisations and bodies). Public services account for 18.2% of total gross value added, the other excluded industries for 7.8%.

situation, demand, sales, and orders as most important for determining their business situation, while economic policy and industry or economy-wide sentiments are considered less relevant. The responses are summarized as balance statistics, which are calculated as the difference in the percentage shares of the responses "good" and "poor".

The main advantage of the survey-based measures is that they are available on a monthly basis and with no publication lag. Table S2 shows that traditional activity measures (gross value added and turnover) are only published either on a quarterly basis (if at all), or with a substantial lag. During a pandemic, where timely data availability is paramount for real-time analyses and decision-making under uncertainty, gross value added and turnover are inferior compared to the business situation from the ifo Business Survey.

This notwithstanding, the survey-based measures must show a high correlation with the traditional measures of activity provided by the Federal Statistical Office of Germany (17). To show this, we run the following regressions for each industry j:

$$\Delta \ln(Y_{j,t}) = c_0 + c_1 \Delta B_{j,t} + \varepsilon_{j,t},$$

where $B_{j,t}$ denotes the ifo business situation and $Y_{j,t}$ gross value added in quarter t. Industries are aggregated to the level of economic sections (i.e. the one-digit level). The results of the regressions are summarized in Table S3; Figure S5 visualizes the results for the first column, i.e. the overall economy.[¶] Most of the elasticities of gross value added with respect to the ifo business situation (c_1) are positive and statistically significant, implying that changes in the business situation are sufficiently precise and timely indicators for current output changes.

While $y^{b,j}$ is constant across all scenarios, $y^{s,j}$ is allowed to vary. We start by normalizing the activity level of the model prior to the shutdown on March 19th to zero. The activity prior to the shutdown refers to the average of the balance statistics of the business situation in January and February 2020. We refer to this as the baseline business situation. To obtain an estimate of $y^{b,j}$, for each industry, we first compute the difference between the balance statistic of the business situation during the shutdown in April 2020 and the baseline business situation (see Table S4). We then apply the following transformation to the balance point differences, ensuring that $0 \leq y^{b,j} \leq 100$:

$$y^{b,j} = \frac{x^{b,j} + 200}{200} \times 100,$$

where $x^{b,j}$ are the balance point differences. Note that the balance point differences are not meant to reflect absolute differences of gross value added. Instead, they indicate the relative degree to which industries are hit during the shutdown and how they perform thereafter. The transformation such that $y^{b,j}$ is between 0 and 100 captures the intuition that economic capacity can range from zero to full capacity.

The activity level after the introduction of the new policy, $y^{s,j}$, is estimated in several steps. We first calculate the difference between the balance statistic of the business situation in June 2020 and the baseline business situation (see Table S4), and again apply the transformation described above. This yields the change in economic activity that is associated with the gradual lifting of the shutdown measured in Germany after April 20th. We obtain two corresponding R_{t} values from the SECIR model, one referring to the reproduction number before ($R_{\text{t}} = 0.53$) and one after the lifting ($R_{\text{t}} = 0.85$). To obtain estimates for $y^{s,j}$ for all values of R_{t}, we assume the relationships between the observed change in R_{t} and the industry-specific changes in economic activity to be linear. For instance, in a scenario where we simulate an increase of R_{t} that is twice as much compared to the observed change from 0.53 to 0.85, economic activity in each industry also increases twice as much.

Note that in the case of Germany, not all industries were affected exogenously by the shutdown measures in the sense that the measures were imposed by the government. For instance, the shutdown of businesses in mid-March did not apply to manufacturing firms, yet we observe a drop in production of these firms. This is due to an endogenous reaction to sluggish demand, disrupted supply chains, or a shortage in labor supply. We take this into account and distinguish between exogenous and endogenous industries (see Table S4). For *exogenous* industries, $y^{s,j}$ is estimated as described above. For *endogenous* industries, we calculate the activity level based on an input-output matrix, which specifies to what extent the production in one industry is affected by changes in production in another industry (18). We use the input-output matrix to calculate the change in activity level for each endogenous industry, Δy_i, based on the changes in activity levels in all exogenous industries:

$$\Delta y_i = \frac{1}{\alpha_i} \sum_{j=1}^{J} \Delta y_j \alpha_j l_{ij} I_j^e,$$

for $i \neq j$. l_{ij} specifies the change in output in industry i that is due to a one unit change in output in industry j. α_i and α_j are the respective industry's shares in total economic output. I_j^e is an indicator variable equal to one if industry j is an exogenous industry, and zero otherwise. We additionally assume the shutdown duration to be constant across all endogenous industries and scenarios. Specifically, we set the shutdown duration $R^{s,j} - S = 30$, i.e. the duration in the reference scenario.

Finally, the estimate for $r^{s,j}$, the duration of the recovery period of industry j in scenario s, is based on a special question in the ifo Business Survey in May. Respondents were asked about the expected duration until their business situation would return to normal once the shutdown measures were lifted. For the reference scenario ($R_{\text{t}} = 0.53$), we take the mean of these expectations for each industry, as well as the mean of their expected best and worst case durations for robustness tests (see Table S4). Similar to $y^{s,j}$, all other scenarios assume a linear relationship between R_{t} and $r^{s,j}$. To estimate the linear

[¶] While gross value added measures economic activity of all industry sections from A to U, the ifo Business Survey only covers roughly three quarters of the total economy.

relationship between R_t and the recovery time, we assume that in the scenario with $R_t = 0.85$ it takes the firms two months less to fully recover. For all scenarios (including the reference scenario), we aggregate the recovery durations to a weekly frequency to prevent weekday effects (i.e. the changes in durations between the scenarios are always multiples of seven days). For the endogenous industries, we assume the recovery periods to be constant across scenarios (but not across industries) and set the recovery duration equal to the durations in the reference scenario.

Robustness tests. To evaluate the robustness of our baseline result, we run a battery of sensitivity tests where we individually vary each model parameter. The results are shown in Table S5. Overall, we find that the baseline result is highly robust, confirming our main finding. In all robustness tests, costs are lowest in the scenarios with slight, step-wise loosening. The minima are all between R_t values of 0.7 and 0.8. Thus, from an economic point of view, a tightening as well as a too strong loosening of the shutdown measures is not the optimal strategy.

In the first two robustness tests, we vary the linear-relationship assumption between the reproduction number and economic activity (columns 1 and 2 in Table S5). We re-scale the estimated coefficient slope for each industry by the factors 2 and 0.5, respectively. That is, we assume that the change in activity when changing the reproduction number is double (half) in magnitude compared to the baseline. Similarly, we vary the linear-relationship assumption between the reproduction number and the duration of the recovery period (columns 2 and 3). Again, we re-scale the coefficient slope by the factor 2 (0.5), i.e. it takes each industry double (half) the time to fully recover from the shutdown.

Further robustness tests vary the assumptions about the shutdown duration or the exogenously affected industries. First, we change the threshold of new cases per day from 300 to 200 and 400, respectively (columns 5 and 6). This captures the intuition that policy-makers might aim at lower or higher daily case numbers. Second, since the estimated duration to reach 300 daily cases for each R_t value is subject to sampling uncertainty, we additionally calibrate our model using the 2.5^{th} and 97.5^{th} percentile of the distribution, i.e. assuming that it took less and more days to reach the 300 cases (columns 7 and 8). Third, we specify all service industries (sections J to Q in Table S4) to be exogenously affected by the shutdown measures (column 9). This controls for the potential issue that the government might have more control over economic activity than we assume in the baseline. Finally, we vary the period when a vaccine becomes available at large scale by shifting the date forward and backward by 120 days compared to the baseline (columns 10 and 11).

In two final robustness tests, we adjust the recovery durations in each section (see Table S4). Instead of relying on the mean of the expected (likeliest) duration, we use the expected best- and worst-case durations (columns 12 and 13).

Isocost curves. The assumption of a linear relationship between the reproduction number and economic activity is arguably a strong one. To test whether our result crucially depends on this assumption, we calculate isocost curves for the baseline scenario. For each reproduction number, the isocost curve specifies the activity level that would be required such that the resulting costs are equal to the costs of the reference scenario ($R_t = 0.53$).

The results are shown in Figure S6. For each economic section, the black line represents the assumed linear relationship, and the red line the isocost curve. For $R_t < 0.53$, economic activity would have to *increase* in order to yield the same costs as the reference scenario. Intuitively, the recovery duration increases with lower values of R_t, but the reduction in the shutdown period is not sufficient to compensate. It is unlikely that a more restrictive shutdown leads to an increase in economic activity, thus strengthening our finding that further tightening the measures leads to higher costs.

For $R_t > 0.53$, the results are twofold. For reproduction numbers slightly above the reference scenario, activity would have to *fall* to yield the same costs. Again, it is unlikely that this is the case in reality. For $R_t > 0.85$, the activities would have to be significantly higher than linearity in sections H, I, and $R-U$. Taken at face value, this would indicate that the optimal reproduction number might be higher than what we find in our baseline scenario. However, it is more likely that the non-linearity goes in the other direction, i.e. that there are diminishing returns to loosening the shutdown, such that the activity levels would lie *below* linearity for R_t values close to one. In fact, diminishing returns would speak even more in favour of our baseline result.

International development and industry-specific export shares. Foreign countries may affect the economy in Germany either via the activity level during the shutdown, or via the recovery path thereafter. Effects on the former are captured by the ifo Business Survey by default, as respondents are asked about their own business activity, taking into account the economic conditions of their trading partners.[‖] In our baseline specification, we calibrate the recovery duration for each sector by exploiting the respondents' expected time until their firm has fully recovered after the lockdown. Their answer reflects their expectation about economic conditions in foreign countries.

To get an intuition about the relevance of foreign countries for our results and potential effects not captured by the ifo Business Survey, we calculate the export shares for each section (see Figure S7, panel **(A)**). The shares are calibrated via the Leontief input-output matrix and calculated as the share of each section's exports to their sum of household and government consumption, investment, and exports (18). While most exogenous industries, which are directly affected by domestic shutdown policies, only have little export shares and are thus less affected by the economic conditions in foreign countries, the endogenous industries mainly consist of firms from the export-oriented German manufacturing industries. The shutdown and recovery durations for endogenous sectors are held constant across the different scenarios to retain comparability. Thus, the economic activity in the endogenous (including export-heavy) industries in our scenarios are only indirectly affected by domestic policy

‖ In the April survey (starting point of our policy scenarios), we consider responses of all managers to estimate the initial economic shutdown situation in several industries. However, to calibrate the relationship between domestic opening policies and economic activity, we only use responses of firm managers of the exogenous sectors in the second survey in June.

measures (via domestic input-output-linkages in the supply chain). We explicitly control for the effect of the international developments of the pandemic and shutdown restrictions abroad.

As most exogenous sectors only have little export shares within their sectors (except e.g. tourism), they are less affected by the economic conditions in foreign countries. Note that although e.g. section H has a rather high export share of almost 40%, the section is small compared to e.g. section C in terms of economic output. Panel **(B)** therefore additionally shows the shares for each section's exports to the overall output of the total economy. In total, exports of the exogenous sections contribute 4.8% percent to the overall output in Germany, whereas exports of endogenous sections contribute by 26.9%. Thus, our simulation model controls for around 95 percent of overall economic output (18).

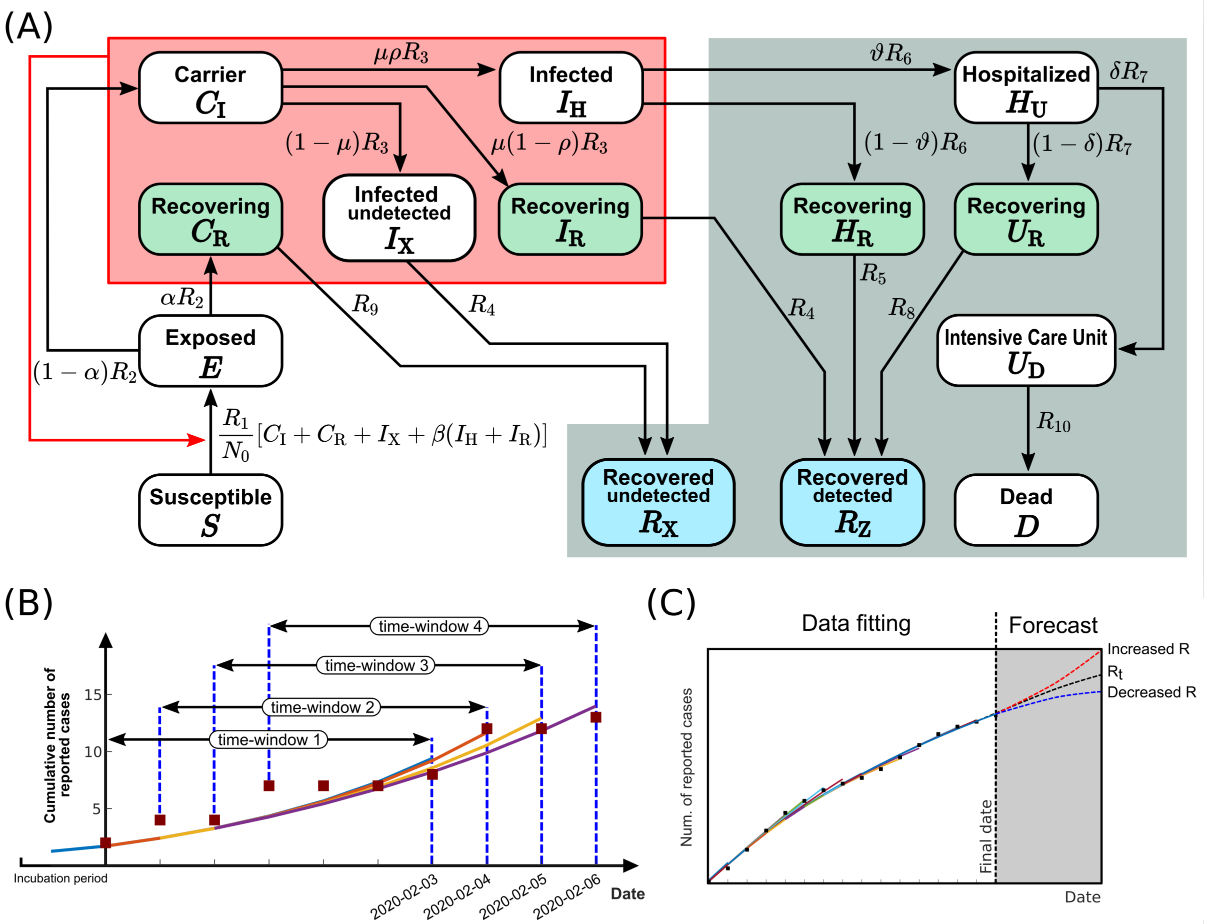

Fig. S1. (A) The scheme of the SECIR model. The model distinguishes susceptible (S), healthy individuals without immune memory of CoV, exposed (E), who carry the virus but are not yet infectious to others, carriers ($C_{I,R}$), who carry the virus and are infectious to others but do not yet show symptoms, infected ($I_{H,R,X}$), who carry the virus with symptoms and are infectious to others, hospitalized ($H_{U,R}$), who experience a severe development of the disease, transferred to intensive care unit ($U_{R,D}$), dead (D), and recovered ($R_{D,X}$), who acquired immune memory and cannot be infected again. Recovery happens from each of the states C_R, I_X, I_R, H_R, U_R. See Table S1 for parameter values. (B) Algorithm of calculating time-varying reproduction number with sliding time-window. Starting from an exposed population based on the initial case reports, the parameter R_1 was fitted to the 1-week time window in the data and the corresponding R_t value was calculated. Next, starting from the state condition of the model at the first time-point, the fitting process was repeated for the time-window shifted by 1 day. This process was repeated for the whole duration of the epidemic. The calculated R_t value was reported for the final date of each time-window. (C) Scheme of prospective simulations. Time evolution of the model variables was obtained from the case reports until the starting date of the prospective study. Then, starting from the last state condition of the model, the numerical simulation was continued with imposed fixed values of R_1 that correspond to the R_t values of interest.

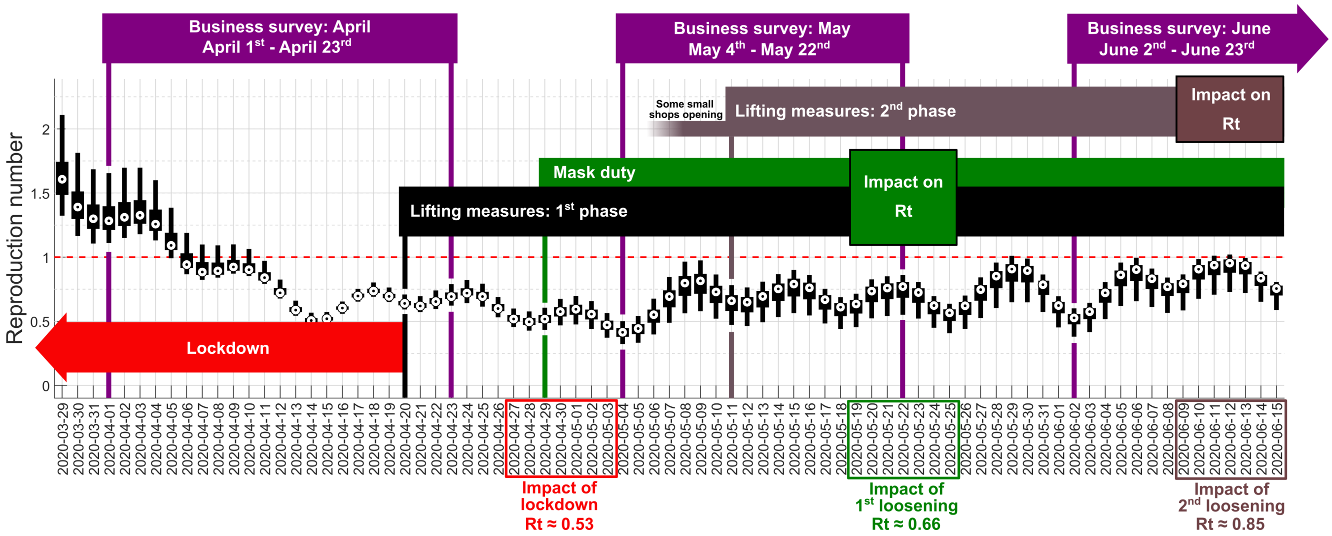

Fig. S2. Time evolution of reproduction number in Germany. The timeline of business surveys and NPIs are marked. The time-windows used for pooling R_t values associated with each NPI is shown on the horizontal axis. The boxplots illustrate the median, 25- and 75-percentiles, maximum and minimum values.

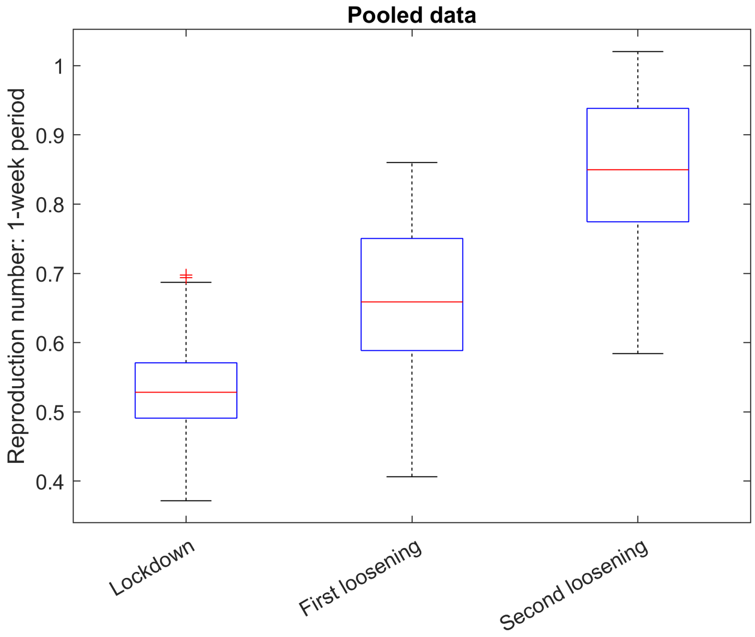

Fig. S3. Distribution of R_{t} values associated with NPIs. Each scenario and corresponding time-window for pooling the data are shown in Fig. S2. The boxplots illustrate the median, 25- and 75-percentiles, maximum and minimum values. The median was used for the economical model.

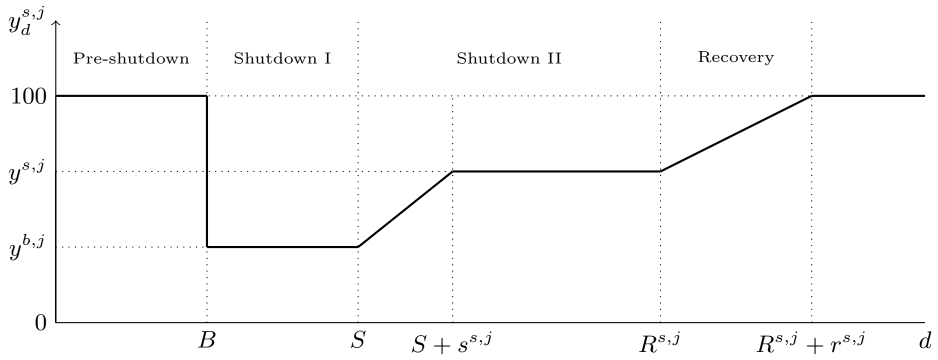

Fig. S4. The figure illustrates the process of economic activity in the model. Starting from a pre-shutdown level, the economy experiences a decline in activity during the shutdown (from 100 to $y^{b,j}$). While the measures are in place, the policy-makers may adjust their severity (from $y^{b,j}$ to $y^{s,j}$). During the recovery phase, the economy slowly returns to its pre-shutdown level (from $y^{s,j}$ to 100).

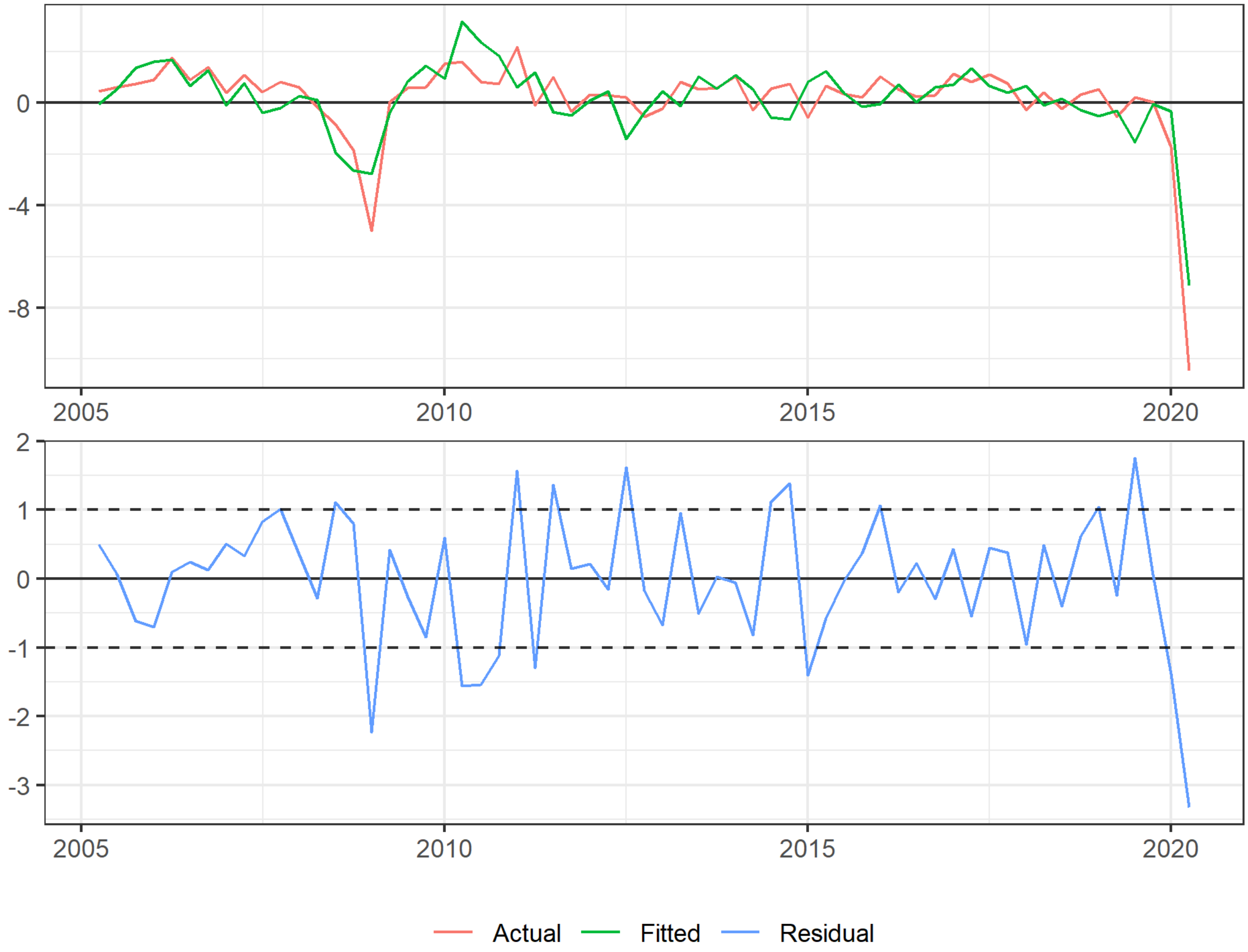

Fig. S5. Regression output

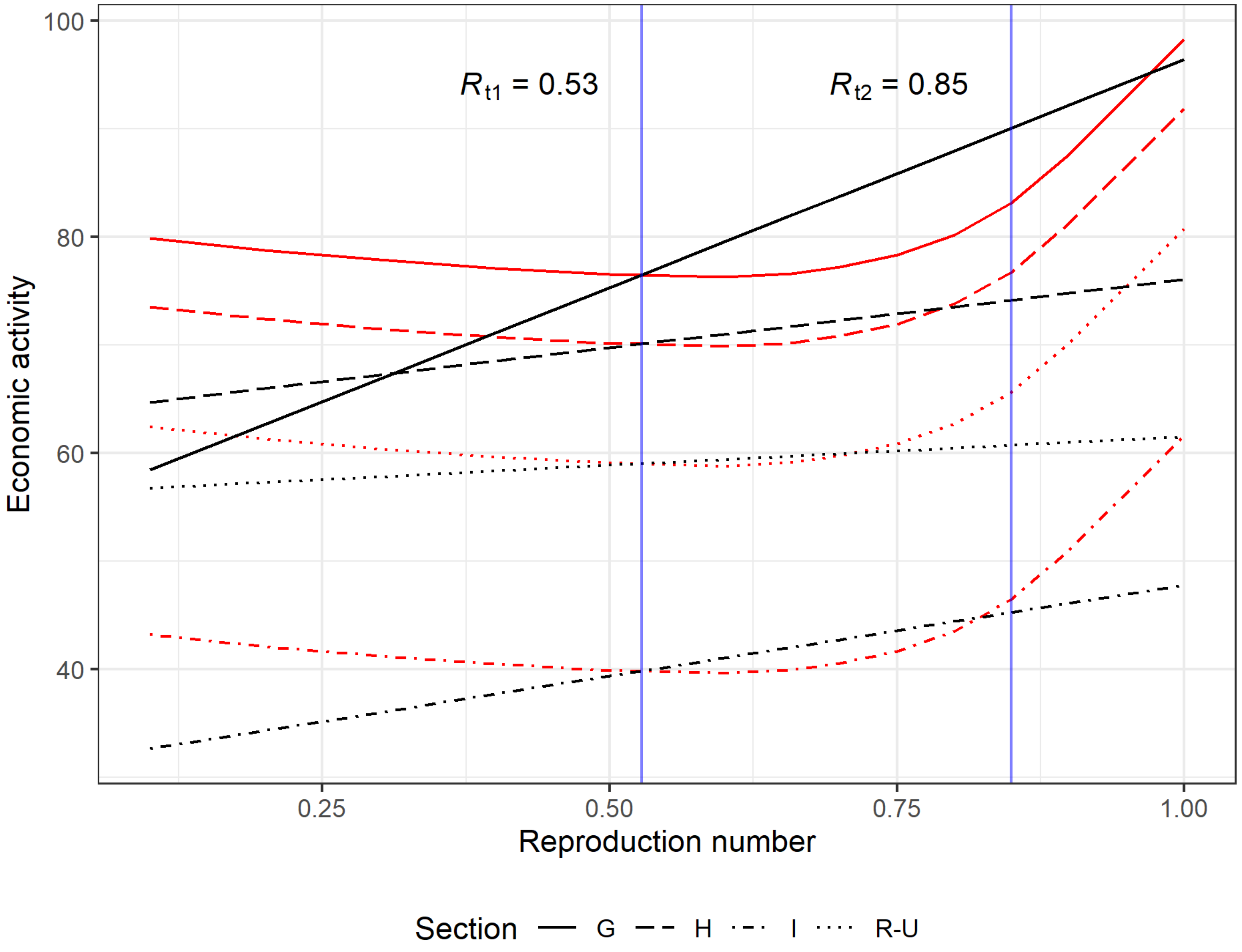

Fig. S6. The black lines show the linear relationships between changes in industry-specific economic activity and changes in the reproduction number. The vertical blue lines indicate the R_t values 0.53 and 0.85 that are used to estimate the slope. The red lines are isocost curves and specify the activity level that would be required such that the resulting costs are equal to the costs of the reference scenario ($R_t = 0.53$)

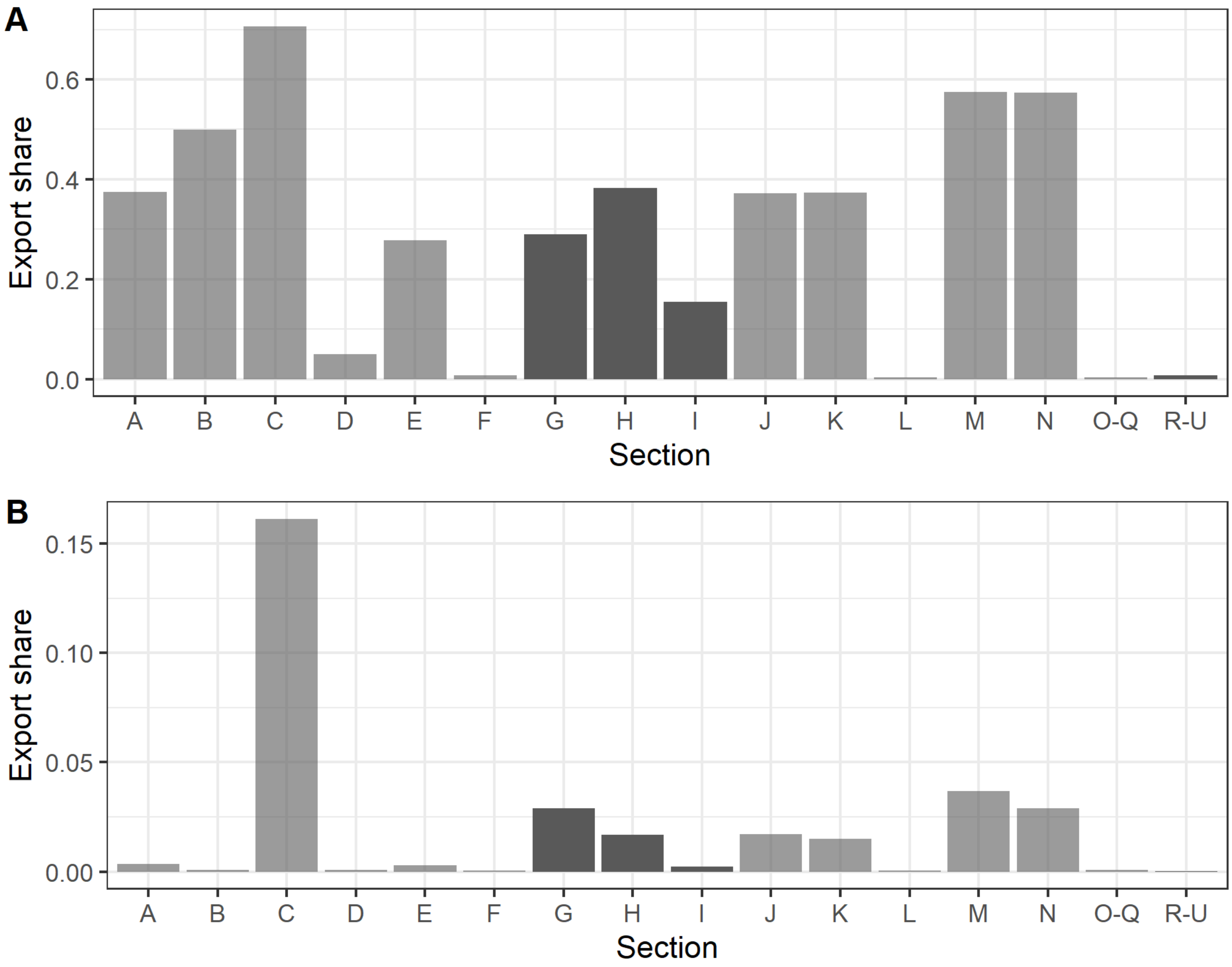

Fig. S7. Panel $(\mathbf{A})$ shows the export shares for each section. The shares are calculated as the share of each section's exports to their sum of household and government consumption, investment, and exports. Panel $(\mathbf{B})$ shows the shares for each section's exports to overall output. Exogenous sections are indicated by dark-grey bars, and endogenous sections by light-grey bars. The data is taken from the Leontief input-output matrix (18)

Table S1. Parameter sets of the SECIR model.

Parameter	References	Ranges	
		Minimum	Maximum
R_1	Variable; fitted.		
R_2	$1/R_2 = 5.2 - 1/R_3$; median incubation period is 5.2 days (19).		
R_3	(20, 21)	$\frac{1}{4.2}$	$\frac{2}{5.2}$
R_4	(22)	$\frac{1}{14}$	$\frac{1}{4}$
R_5	(23, 24)	$\frac{1}{16}$	$\frac{1}{7}$
R_6	(25, 26)	$\frac{1}{7}$	$\frac{1}{2.5}$
R_7	(23, 27)	$\frac{1}{14}$	$\frac{1}{4}$
R_8	(24)	$\frac{1}{16}$	$\frac{1}{5}$
R_9	$\frac{1}{R_9} = \frac{1}{R_3} + \left(0.5 \times \frac{1}{R_4}\right)$		
R_{10}	(23)	$\frac{1}{7.5}$	$\frac{1}{3.5}$
δ_0	(24, 28)	0.15	0.77
α	(29)	0.01	0.5
β	Assumed	0.05	1
ρ_0	(28, 30)	0.1	0.35
ϑ_0	(23, 28)	0.15	0.4
μ	Assumed; fixed value $\mu = 1$.		

Table S2. Data Availability

	Section								
	C	F	G	H	I	J	L	M-N	R-T
**Gross Value Added**									
Frequency	q	q	q	q	q	q	q	q	q
Publication lag	$T + 55$	$T + 55$	$T + 55$	$T + 55$	$T + 55$	$T + 55$	$T + 55$	$T + 55$	$T + 55$
**Turnover**									
Frequency	m	m	m/q	q	m	q	-	q	-
Publication lag	$T + 35$	$T + 70$	$T + 60$ / $T + 60$	$T + 70$	$T + 60$	$T + 70$	-	$T + 70$	-
**ifo Business Situation**									
Frequency	m	m	m	m	m	m	m	m	m
Publication lag	$T - 5$	$T - 5$	$T - 5$	$T - 5$	$T - 5$	$T - 5$	$T - 5$	$T - 5$	$T - 5$

Note: The frequency with which the data is published is either monthly (m) or quarterly (q). $T + n$ denotes the publication lag, where T is the month / quarter to which the indicator refers to, and n the days after the end of the month / quarter. The ifo Business Survey is conducted in the first half of each month and is published about five days before the end of the month.

Table S3. Regression results

j	A-U	C	F	G	H	I	J	L	M,N	R,S,T
c_0	0.21^{*}	0.16	-0.13	0.39^{**}	0.25	-0.50	1.14^{***}	0.26^{**}	0.22	-0.24
	(0.12)	(0.15)	(0.19)	(0.15)	(0.22)	(0.65)	(0.21)	(0.11)	(0.18)	(0.23)
c_1	0.19^{***}	0.26^{***}	0.14^{***}	0.12^{***}	0.13^{***}	0.69^{***}	0.11^{***}	0.01	0.25^{***}	0.30
	(0.02)	(0.04)	(0.05)	(0.03)	(0.04)	(0.17)	(0.03)	(0.02)	(0.06)	(0.19)
Obs.	61	117	117	117	61	61	61	61	61	61
R^2	0.68	0.59	0.05	0.16	0.42	0.74	0.18	0.00	0.59	0.36

Note: The sample period ranges from the second quarter 1991 (2005) to the second quarter 2020. Heteroskedasticity- and autocorrelation-consistent standard errors are shown in parentheses. *** / ** / * denotes significance at the 1 / 5 / 10 % level.

Table S4. Model Assumptions

Section	Name	α	Activity: April	Activity: June	Recovery	Recovery: best	Recovery: worst	Exogenous (yes / no)
A	Agriculture, forestry and fishing	0.9						
B	Mining and quarrying	0.1						
C	Manufacturing	22.8	-18.8	-24.3	9.2	5.2	16.1	0
D	Electricity, gas, steam and air conditioning supply	1.7						
E	Water supply; sewerage, waste management and remediation activities	1.1						
F	Construction	4.7	-8.5	-9.8	9.4	4.5	15.5	0
G	Wholesale and retail trade; repair of motor vehicles and motorcycles	10.0	-23.5	-9.9	8.4	5.0	14.7	1
H	Transportation and storage	4.4	-29.9	-25.9	9.1	5.1	16.5	1
I	Accommodation and food service activities	1.6	-60.2	-54.8	10.5	6.3	17.5	1
J	Information and communication	4.6	-25.3	-19.1	8.2	4.4	14.9	0
K	Financial and insurance activities	4.0						
L	Real estate activities	10.6	-12.7	-11.3	9.0	5.1	16.0	0
M	Professional, scientific and technical activities	6.4	-19.0	-14.0	8.9	5.1	15.8	0
N	Administrative and support service activities	5.1	-35.2	-31.1	8.9	5.1	15.8	0
O,P,Q	Public administration and defence; compulsory social security; Education; Human health and social work activities	18.2						
R,S,T,U	Arts, entertainment and recreation; Other service activities; Activities of households as employers; undifferentiated goods- and services-producing activities of households for own use; Activities of extraterritorial organisations and bodies	3.8	-41.0	-39.3	8.8	5.0	15.3	1

Note: The table shows the model assumptions for each economic section of the NACE Rev. 2 classification. Column three with the header α shows each section's share in total economic output. Columns four and five show economic activity in April and June relative to the pre-shutdown level, which is normalized to zero. Sections A, B, D, E, O, P, and Q are set to zero due to the lack of coverage in the ifo Business Survey. Column six shows the recovery duration (in months) that it takes each industry within a section to return to its pre-shutdown level. Column seven and eight show the best-case and worst-case recovery durations, respectively. Column nine indicates which sections are specified to be exogenous.

Table S5. Robustness Tests

R_t	Relative costs													
	Baseline	Robustness tests												
		1	2	3	4	5	6	7	8	9	10	11	12	13
0.10	17.6	31.2	10.8	12.5	28.0	16.8	18.1	17.8	16.9	27.6	17.6	17.6	19.2	16.8
0.20	12.6	22.7	7.6	8.9	20.2	12.0	13.2	12.9	12.2	19.8	12.6	12.6	13.4	12.3
0.30	8.2	14.9	4.8	5.8	13.2	7.9	8.6	8.2	7.8	12.6	8.2	8.2	8.9	8.2
0.40	4.3	8.0	2.5	3.0	6.8	4.2	4.7	4.3	3.9	6.5	4.3	4.3	4.0	4.3
0.50	0.8	1.6	0.4	0.7	1.5	0.8	0.8	0.8	0.7	1.1	0.8	0.8	1.1	0.9
0.53	0.0	0.0	0.0	0.0	0.0	0.0	0.0	0.0	0.0	0.0	0.0	0.0	0.0	0.0
0.60	-2.2	-4.2	-1.2	-1.5	-3.4	-1.9	-2.2	-2.4	-2.3	-3.5	-2.2	-2.2	-2.2	-2.4
0.66	-3.5	-7.2	-1.7	-2.3	-5.5	-3.1	-3.8	-3.7	-3.3	-5.2	-3.5	-3.5	-2.8	-3.8
0.70	-4.2	-9.1	-1.7	-2.9	-6.8	-3.6	-4.5	-4.3	-3.7	-6.1	-4.2	-4.2	-3.3	-4.7
0.75	-4.6	-11.2	-1.3	-3.0	-7.8	-3.6	-5.1	-4.8	-4.0	-6.4	-4.6	-4.6	-3.5	-5.8
0.80	-4.3	-12.9	0.0	-2.4	-8.0	-3.0	-5.2	-4.7	-3.4	-4.8	-4.3	-4.3	-2.2	-6.2
0.85	-2.7	-11.7	3.0	-0.7	-6.9	-0.9	-4.0	-3.2	-1.3	-0.7	-2.7	-2.7	0.9	-5.6
0.90	2.0	-7.6	10.0	4.3	-2.5	4.9	-0.2	1.4	4.2	9.5	2.0	2.0	8.3	-3.0
1.00	31.3	16.8	52.9	33.9	26.4	29.9	32.6	31.6	30.6	74.0	17.7	45.0	51.4	15.0

Note: The table shows shows the relative costs for the baseline and the robustness tests. Relative costs are given as the percentage differences in total loss of economic activity compared to the reference scenario ($R_t = 0.53$). In columns 1 and 2, we vary the linear-relationship assumption between the reproduction number and economic activity. In columns 3 and 4, we vary the linear-relationship assumption between the reproduction number and the duration of the recovery period. In columns 5 and 6, we change the threshold of new cases per day from 300 to 200 and 400, respectively. In columns 7 and 8, we calibrate the model using the 2.5^{th} and 97.5^{th} percentile of the duration distribution. In column 9, we specify all service industries to be exogenously affected by the shutdown measures. In columns 10 and 11, we vary the period when a vaccine becomes available at large scale by shifting the date forward and backward by 120 days compared to the baseline. In columns 12 and 13, we use the expected best- and worst-case recovery durations instead of the mean of the expected (likeliest) duration.

References

1. T Wollmershäuser, et al., ifo Konjunkturprognose Sommer 2020: Deutsche Wirtschaft – es geht wieder aufwärts. *ifo Schnelldienst* **73**, 3–58 (2020).
2. Genesis-Online, Bevölkerung, Stichtag (2020).
3. John Hopkins University, COVID-19 Dashboard by the Center for Systems Science and Engineering (CSSE) at Johns Hopkins University (JHU) (2020).
4. Human Mortality Database, Sweden, Population size (abridged) (2020).
5. Human Mortality Database, United Kingdom, Population size (abridged) (2020).
6. Human Mortality Database, The United States of America, Population size (abridged) (2020).
7. K Prem, et al., The effect of control strategies to reduce social mixing on outcomes of the COVID-19 epidemic in Wuhan, China: a modelling study. *The Lancet Public Heal.*, e261–e270 (2020).
8. N Hoertel, et al., A stochastic agent-based model of the SARS-CoV-2 epidemic in France. *Nat. Medicine*, 1–5 (2020).
9. F Ndaïrou, I Area, JJ Nieto, DFM Torres, Mathematical modeling of COVID-19 transmission dynamics with a case study of Wuhan. *Chaos Solitons Factals* **135**, 109846 (2020).
10. S Khailaie, et al., Development of the reproduction number from coronavirus SARS-CoV-2 case data in Germany and implications for political measures. *BMC Medicine* **19** (2021).
11. P Vanella, et al., Pitfalls and solutions in case fatality risk estimation - a multi-country analysis on the effects of demographics, surveillance, time lags between reports and deaths and healthcare system capacity on COVID-19 CFR estimates. *Vienna Yearb. Popul. Res.* **20** **[forthcoming]** (2022).
12. O Diekmann, JA Heesterbeek, JA Metz, On the definition and the computation of the basic reproduction ratio r0 in models for infectious diseases in heterogeneous populations. *J Math Biol* **28**, 365–382 (1990).
13. O Diekmann, JAP Heesterbeek, MG Roberts, The construction of next-generation matrices for compartmental epidemic models. *J R Soc Interface* **7**, 873–885 (2010).
14. JC Lagarias, JA Reeds, MH Wright, PE Wright, Convergence properties of the nelder-mead simplex method in low dimensions. *SIAM J Optim.* **9**, 112–147 (1998).
15. S Sauer, K Wohlrabe, *ifo Handbuch der Konjunkturumfragen.* (ifo Beiträge zur Wirtschaftsforschung) No. 88, (2020).
16. S Lautenbacher, Subjective uncertainty, expectations, and firm behavior. *MPRA Work. Pap.* **103516** (2020).
17. R Lehmann, The forecasting power of the ifo business survey. *CESifo Work. Pap.* **8291** (2020).
18. Federal Statistical Office, *Volkswirtschaftliche Gesamtrechnungen. Input-Output-Rechnung nach 12 Gütergruppen / Wirtschafts- und Produktionsbereichen.* (Federal Statistical Office of Germany), (2020).
19. Q Li, et al., Early transmission dynamics in Wuhan, China, of novel coronavirus-infected pneumonia. *N Engl J Med* **382**, 1199–1207 (2020).
20. H Nishiura, NM Linton, AR Akhmetzhanov, Serial interval of novel coronavirus (COVID-19) infections. *Int J Infect Dis* **93**, 284–286 (2020).
21. S Zhao, et al., Estimating the serial interval of the novel coronavirus disease (COVID-19): A statistical analysis using the public data in Hong Kong from January 16 to February 15, 2020. *medRxiv* (2020).
22. R Woelfel, et al., Clinical presentation and virological assessment of hospitalized cases of coronavirus disease 2019 in a travel-associated transmission cluster. *medRxiv* (2020).
23. D Wang, et al., Clinical characteristics of 138 hospitalized patients with 2019 novel coronavirus-infected pneumonia in Wuhan, China. *JAMA* (2020).
24. World Health Organization, Report of the WHO-China joint mission on coronavirus disease 2019 (COVID-19) (2020).
25. Q Cai, et al., 2019-nCoV pneumonia in a normal work infectious diseases hospital besides Hubei province, China. *SSRN J.* (2020).
26. WJ Guan, et al., Clinical characteristics of coronavirus disease 2019 in China. *N Engl J Med* (2020).
27. Robert Koch Institut, Steckbrief zur Coronavirus-Krankheit-2019 (COVID-19) (2020).
28. Novel Coronavirus Pneumonia Emergency Response Epidemiology Team, The epidemiological characteristics of an outbreak of 2019 novel coronavirus diseases (COVID-19) in China. *Zhonghua Liu Xing Bing Xue Za Zhi* **41**, 145–151 (2020).
29. Istituto Superiore di Sanità, Report sulle caratteristiche dei pazienti deceduti positivi a COVID-19 in Italia. il presente report è basato sui dati aggiornati al 20 marzo 2020. (2020).
30. R Verity, et al., Estimates of the severity of COVID-19 disease. *medRxiv* (2020).
